# Phylogenetic evidence for extensive lateral acquisition of cellular genes by Nucleocytoplasmic large DNA viruses

**DOI:** 10.1186/1471-2148-8-320

**Published:** 2008-11-26

**Authors:** Jonathan Filée, Noëlle Pouget, Mick Chandler

**Affiliations:** 1 Laboratoire de Microbiologie et Génétique Moléculaires, C.N.R.S, Campus Toulouse III, 118 Route de Narbonne, F-31062 Toulouse Cedex, France

## Abstract

**Background:**

Nucleo-Cytoplasmic Large DNA viruses (NCLDV), a diverse group that infects a wide range of eukaryotic hosts, exhibit a large heterogeneity in genome size (between 100 kb and 1.2 Mb) but have been suggested to form a monophyletic group on the basis of a small subset of approximately 30 conserved genes. NCLDV were proposed to have evolved by simplification from cellular organism although some of the giant NCLDV have clearly grown by gene accretion from a bacterial origin.

**Results:**

We demonstrate here that many NCLDV lineages appear to have undergone frequent gene exchange in two different ways. Viruses which infect protists directly (Mimivirus) or algae which exist as intracellular protists symbionts (Phycodnaviruses) acquire genes from a bacterial source. Metazoan viruses such as the Poxviruses show a predominant acquisition of host genes. In both cases, the laterally acquired genes show a strong tendency to be positioned at the tip of the genome. Surprisingly, several core genes believed to be ancestral in the family appear to have undergone lateral gene transfers, suggesting that the NCLDV ancestor might have had a smaller genome than previously believed. Moreover, our data show that the larger the genome, the higher is the number of laterally acquired genes. This pattern is incompatible with a genome reduction from a cellular ancestor.

**Conclusion:**

We propose that the NCLDV viruses have evolved by significant growth of a simple DNA virus by gene acquisition from cellular sources.

## Background

DNA viruses are ubiquitous components of the biosphere and their number exceeds that of cells by at least an order of magnitude [1]. This abundance is also accompanied by an extraordinary diversity in genome size, composition and organisation [2] and viruses have been divided into many different classes based on these criteria. These observations raise multiple questions about the origin and evolution of different viral classes.

Briefly, three main hypotheses have been proposed to explain the emergence of DNA viruses:

- The "*cell degeneration" *hypothesis advocates that viruses derive from a cellular ancestor via progressive simplification [3].

- The traditional "*escape hypothesis*" postulates that viruses are autonomous genetic elements that have escaped from a cellular genome [4].

- The "*virus first*" hypothesis proposes that viruses are descendants of primordial genetic elements that were components of the primitive soup [5].

These hypotheses have been extensively discussed in the light of recent advances in comparative genomics (see for example [6] and [7] and references therein). Interestingly, the idea that some DNA viruses originate by reductive evolution from a cellular ancestor was boosted by the discovery of a giant eukaryotic virus: the Mimivirus [8]. Mimivirus has a genome of 1.2 Mb, significantly larger than that of several bacteria [9].

Under the cellular degeneration hypothesis, the mimivirus could be considered as a "missing link" between virus and cell and it has been suggested that it may represent a fourth domain of life in addition to the Bacteria, Archaea and Eukarya [8]. However, mimivirus is one member of a large family of viruses: the Nucleo-Cytoplasmic Large DNA Viruses (NCLDV). This is an extremely diverse group whose members infect a wide range of eukaryotic hosts including algae (Phycodnaviruses), protists (Mimivirus) and Metazoa (Poxviruses, African Swine Fever Virus, Iridoviruses). NCLDV are characterised by a large heterogeneity in genome size (between 100 kb and 1.2 Mb) and, based on a small set of 30 common homologous (core) genes, are thought to be monophyletic. These mainly encode proteins involved in informational processes and virus structure [10]. Several of these, such as the capsid protein gene, have no homologues in cellular sequences but have distant relatives in other classes of viruses. It has been suggested that these shared genes could be considered as "hallmark viral genes" that derive from an ancient virus world [11].

Controversially, it has been proposed that the giant genome of the Mimivirus resulted from extensive accretion of host derived genes [10,12]. This view is supported by scattered evidence of acquisition of host genes by eukaryotic viruses [10,12-14] and by bacteriophages [15-18]. This "gene pickpocket" viewpoint is reminiscent of Hendrix's "moron hypothesis" (for: acquisition of more and more foreign DNA) of bacteriophage evolution [15].

On the other hand, several hypotheses have proposed that viral genes could be the source of new functions for their host including: (i) invention of DNA by the viral world and subsequent transfer to cellular organisms [19]; (ii) replacement of the bacterial-type DNA replication apparatus of the mitochondria and the chloroplast by a phage-type system [20,21]; (iii) acquisition of a nucleus that derives from a large double strand DNA virus such as a Poxvirus [22,23].

However, accumulating evidence shows that some viral genes are only distantly related to their host counterparts. This is not consistent with the idea of frequent horizontal exchange of these genes between host and virus [17,18,24-26].

Thus, the debate concerning the exact role of lateral gene exchange during evolution of each of the many classes of virus remains open. This question seems particularly relevant for NCLDV because of the large variations in genomic repertoire and size in the different lineages. Indeed, we have recently shown that the genome of the giant representatives of the NCLDV (Mimivirus and Phycodnaviruses) display an unexpected abundance of islands of bacterial-like genes. We hypothesized that these Giant Virus genomes grow in size by successive accretion of bacterial-like genes provided by their hosts that graze on bacteria [27]

In this study, we have undertaken a complete phylogenetic survey of NCLDV genes that have homologs in the cellular genome. We show that NCLDV which infect Metazoa display a large collection of host-derived genes, whereas giant NCLDV which infect (or are "associated" with) protists (see below) derive their "non-viral" genes principally from a bacterial source. Moreover, those phycodnaviruses which infect free-living algae do not carry extensive numbers of bacterial-like genes. Taken together, these results suggest that NCLDV grow by accretion of cellular genes rather than simplify themselves by genome reduction from a complex ancestor.

## Results and discussion

### Distribution of bacterial-like genes in NCLDV

We previously reported that *Chlorella *Phycodnaviruses and Mimivirus genomes carry respectively 48 to 57 genes and 96 genes that appeared to be unambiguously of bacterial origin We considered a gene to be originated from a bacteria if the sequence is phylogenetically closer to bacteria than other organisms, including other viruses [27]. These genes tended to be clustered in islands towards the extremities of the genomes. The genomes also carried a large number of diverse bacterial-like mobile genetic elements (MGE) such as insertion sequences, inteins, restriction/modification systems and homing endonucleases [27]. Moreover, several consecutive bacterial-like genes showing synteny with identifiable bacteria appeared to have been co-inherited.

Analysis of additional, newly available, *Chlorella *Phycodnavirus genomes gave similar results (Additional file 1). These data strongly support the idea that some giant NCLDV members have acquired a large panel of genes originating from bacteria.

### Bacterial-like genes are less frequent in Phycodnaviruses of free living algae

We suggested that their eukaryotic hosts, which graze on bacteria, could provide the "ecological" niche for viral access to bacterial gene pools. The Phycodnaviruses analysed infect *Chlorellae *which in turn live in symbiosis with *Paramecia *or Heliozoa from the genus *Acanthocystis *while the Mimivirus infects Amoebae directly.

**Figure 1:**
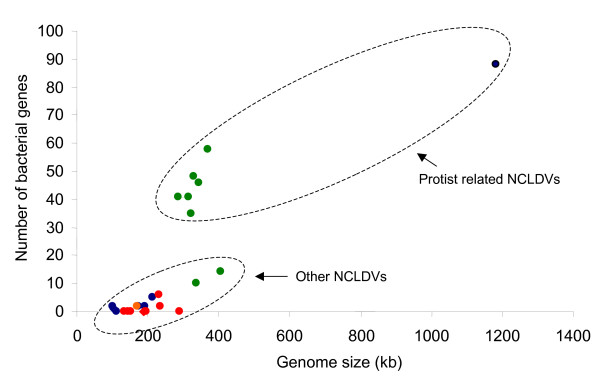
**Number of bacterial-like genes in the NCLDVs**. The number of genes identified as bacterial-like (excluding those of mobile elements) for each virus is plotted as a function of their genome size. Poxviruses are indicated by red circles, Iridoviruses are in blue, the Asfarvirus in orange, Phycodnaviruses in green and the Mimivirus in black.

However, many NCLDV lineages infect metazoa or algae that do not use bacteria as prey. The question which therefore arose was whether these genomes carry a significant number of bacterial-type genes and MGE. We therefore screened a representative set of NCLDV (see Materials and Methods) for such genes and plotted their number against the genome size (Fig. 1). Although there is a general increase in bacterial gene number with genome size, there is also a strong dichotomy between the *Chlorella *Phycodnaviruses and Mimivirus, which are considerably enriched for bacterial genes, and Phycodnaviruses EHV86 and ESV-1 which are not. In addition, very few MGE could be found in these two Algal viruses. Two copies of an intact IS*4*-family element are present in the ESV-1 genome but no inteins restriction/modification systems or bacterial-like homing endonucleases could be found. EHV86 and ESV-1 infect *Emilinia huxleyi *and *Ectocarpus siliculosus *respectively. Importantly these are free-living algae and are not known to ingest bacteria. This provides strong support for lateral bacterial gene transfer since, in contrast to the *Chlorella *viruses and Mimivirus, neither EHV86 nor ESV-1 viruses live in intimate contact with bacteria.

### Bacterial-like genes are rare in metazoan NCLDV

Poxviruses, Iridioviruses and Asfarvirus carry even fewer bacterial-like genes than Phycodnaviruses EHV86 and ESV-1. In Metazoan and viruses from free-living algae, these tend to be scattered in the genome, with no apparent clustering in islands towards the genome ends (data not shown)

As in the case of the two viruses from free-living algae, no or very few MGE were found in Metazoan viruses. Thus, viruses which coexist with protists (i.e. exposed to bacterial foraging) contain a clearly larger proportion of bacterial-like genes compared to metazoan and algal viruses. These data suggest that lateral acquisition of bacterial-like genes and MGE is not a central force in shaping metazoan and algal virus genomes. On the other hand, the genome complexity and size of the Mimivirus and the *Chlorella *Phycodnaviruses seem to be substantially explained by the accretion of a diverse array of bacterial genes and MGE. This important difference in these two classes of NCLDV seems to be a natural consequence of the ecology of their host since, like free living Algae, Metazoa do not (generally) graze on bacteria. This limits genetic promiscuity between the DNA of replicating viruses and that of bacteria.

### Respective abundance of host-derived genes in NCLDV

**Figure 2:**
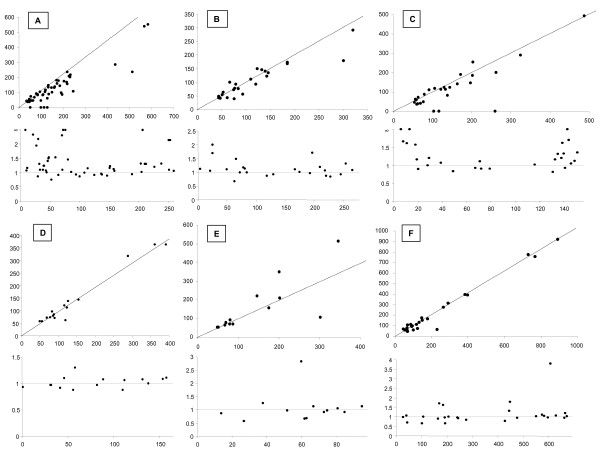
**Distribution of BLAST score for NCLDV ORFs with eukaryotic affinities**. Upper panels: The axes represent the best BLASTP score against the host genome (horizontal) and against a non-redundant (NR) database purged from the closely related sequences of the host. The black lines correspond to equal scores along both axes. Lower panels: The axes represent the ratio: BLAST score against the host divided by the BLAST score against a NR database (vertical) plotted as the genome position of the corresponding ORF. The black lines correspond to the ratio equal to 1. A) Fowlpox BLAST score (best hit in the Bird sequence database against a NR database purged of vertebrate sequences). B) *Amsacta moorei *Poxvirus BLAST score (best hit in the Insect sequence database against a NR database purged of insect sequences). C) Lumpyskin disease Poxvirus (best hit in the *Bos Taurus *sequence database against a NR database purged of vertebrate sequences). D) *Trichoplusia ni *Ascovirus (Iridovirus) (best hit in Insect sequence database against a NR database purged of insect sequences). E) Frog Iridovirus (best hit in the *Xenopus laevis *sequence database against a NR database purged of vertebrate sequences). F) PBCV1 *Chlorella *phycodnavirus (best hit in the green alga and plant sequence database against a NR database purged of green alga and plant sequences).

NCLDV genomes also carry a large set of diverse genes with eukaryotic affinities [10,27]. These could have been inherited from a common ancestor of the NCLDV predating the divergence of the major eukaryotic lineage or could have been acquired by recent lateral gene transfer from the host. Alternatively, the eukaryotic host could have acquired the gene from one of its viruses. Fortunately, these scenarios might be decipherable since the sequences of many of the NCLDV host genomes (or their relatives) are now available. To identify viral genes closely related to those of the host, and therefore constituting good candidates for gene transfer, the following BLAST approach was used:

Viral genes with host homologues were identified using a BLAST search of each viral ORF against the "host" (or related) genome and the best hit score was noted and compared to first hits from a similar BLAST search using a purged non-redundant database (see Materials and Methods). The two corresponding BLAST scores for each gene were then plotted against each other (Fig 2, upper panel). Genes putatively implicated in transfer with the host will appear below the 1:1 diagonal. The ratio "BLAST score host genome/BLAST score NR database" was then calculated for each gene and plotted as a function of the position of that gene on the genome. This analysis enables visualization of the position in the genome of those genes suspected of acquisition from the host since they have a ratio higher than 1 (Fig. 2A–F, lower panel). As we suspect that most laterally transferred genes in NCLDV are acquired by single strand DNA invasion at the tips of the viral genome, we believe that the localisation of the laterally transferred genes in the viral genome could provide additional arguments for identifying host-derived genes as it did in the case of bacterial-like genes [27]. The importance of patterns in viral genome organisation as a source of additional evolutionary arguments has been previously underlined by Shackelton and Holmes [28]

Among the NCLDV, Poxviruses were found to carry a larger proportion of genes with eukaryotic affinities than other members of the group (Additional file 2). An important fraction of these genes is more closely related to the corresponding host homologs than to the other homologs present in a NR database (Fig 2, upper panels: A, B and C). Moreover, genes that are more similar to the host copies tend to be clustered at the tips of the genome (Fig 2 lower panels: A, B and C). To substantiate this effect, the data were fitted with equations describing the two hypotheses: homogeneous distribution along the entire genome or overrepresentation at the tips of the genome. Best fit results were chosen with an F test (P < 0,05). This statistical analysis (Additional file 3) validated the hypothesis that the laterally acquired genes tend to be clustered towards the ends of the poxvirus genomes. This result is in agreement with the hypothesis of multiple lateral gene exchanges with the host.

Iridoviruses and the Asfarvirus have a lower number of genes with eukaryotic affinities (Additional file 2) but a significant fraction of these are also more related to their host homolog than to the other homologs. However, these genes do not have a strong tendency to be present at the tip of their respective genomes but appears randomly distributed along the genome (Additional file 3). This implies that exchange of such genes may have either occurred by a different mechanism than other laterally acquired genes and/or have subsequently undergone rearrangement in the genome.

**Figure 3:**
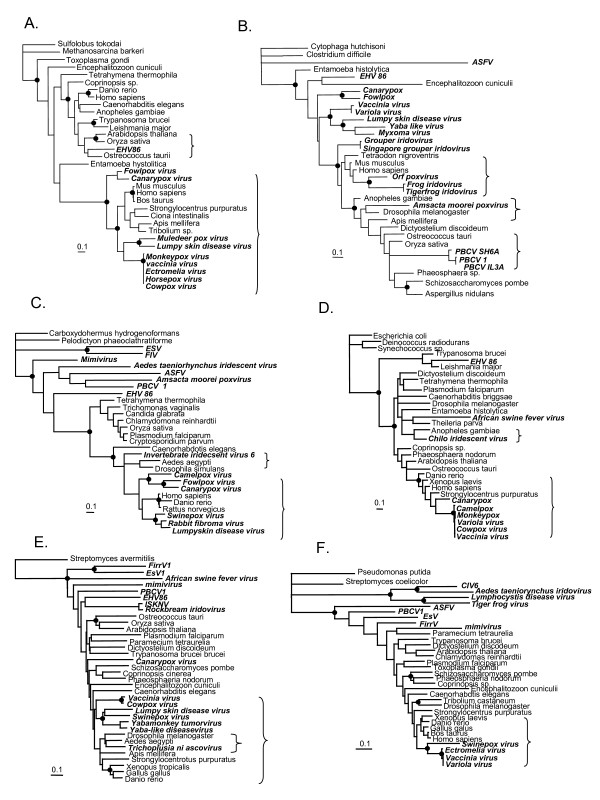
**Maximum likelihood phylogeny of several core NCLDV genes that display lateral gene transfers from the host.** A) ATP-dependant DNA ligase. B) dUTPase. C) Serine/thréonine kinase. D) Thymidine kinase. E) Ribonucleotide reductase (small subunit). F) Ribonucleotide reductase (large subunit). Viral sequences are indicated in italics. Brackets represent putative cases of horizontal gene transfers between the virus and its host. Bootstrap values up to 95% are indicated with black circle. The scale bars represent the number of amino acid substitutions per residue.

Finally, despite having larger genomes, the *Chlorella *Phycodnaviruses and the Mimivirus have the lowest proportion (number/genome length) of genes with potential eukaryotic origins (these numbers are presented in Additional file 2). Only a handful of these eukaryotic-like genes appear to be closely related to their host counterparts (Fig 2 and [26] for the mimivirus). However, the majority of these tend to be present at the tip of the genome. These data indicate that among NCLDV, Poxviruses have the strongest tendency to exchange genes with their host. Iridoviruses and the Asfarvirus seem to be less prone to lateral gene exchange with their host, and the Phycodnaviruses and the Mimivirus, despite having larger genomes, have the lowest number of genes putatively involved in lateral transfers with their respective hosts. In an attempt to validate theses results and to determine the direction of lateral gene transfers between the virus and its host, we reconstructed individual phylogenies of each viral gene that is more similar to its host homolog than to other homologs (some examples are shown in Fig. 3 and discussed below).

### Core genes involved in DNA metabolism have undergone lateral gene transfers from cellular sources

Phylogenies including both viral genes and cellular genes can help to determine the direction of a putative lateral gene transfer. Three scenarios are possible. If the viral genes are ancestral (without any transfer), then all viral genes would be positioned together at the base of the tree and the cellular sequences would be clustered outside the viral groups. If, on the other hand, the viral sequences fall within a cluster of cellular genes, they were probably captured by the virus from the host. Inversely, if the cellular sequences fall into viral clusters, the cellular sequences more probably originated from a virus [28]. The phylogenetic validation of the NCLDV genes showed that all those identified by BLAST to have probably undergone lateral gene exchange with the host are the result of transfers from the host. The results obtained from a selected subset of these genes are presented in Fig. 3. This result shows that a simple and fast BLAST approach permits identification, with reasonable confidence, of viral genes that have been acquired from their host.

Surprisingly, examination of these host-derived genes revealed that several are constituents of the core genes of the NCLDV family [10]. Core genes are thought to be inherited from a common ancestor that predates the divergence of the different lineages of NCLDVs [10]. If this were the case, each of the different NCLDV core genes should cluster together in the phylogenetic tree, ideally at the base of the eukaryotic tree. On the other hand, if some genes were acquired by lateral transfer with the host, the corresponding gene will appear polyphyletic, and ideally, as a sister-taxon of the host sequences. Six core genes follow the last scenario and are therefore probably acquired by polarised lateral gene transfer from the host (Fig 3). They are (Table 1):

**Table 1:** List of core NCLDV genes with cellular homologs and their corresponding phylogenetic status.

Core NCLDV genes	Phylogenetic status of the NCLDVs sequences	Remarks
DNA Polymerase B	Basal	
A18-type helicase	Basal	
Thiol-Oxydoreductase	Basal	
Ser/Thr Kinase	LGT for CIV iridovirus and vertebrate Poxviruses	
PCNA	Basal	
RNR small ssu	LGT for Trichoplusia Ascovirus and mammalian Poxviruses	
RNR large ssu	LGT for mammalian Poxviruses	
Thymidilate kinase	LGT for CIV iridovirus and vertebrate Poxviruses	
Thymidilate synthase ThyA-type	Polyphyletic	Probable displacement of this enzyme in the Chlorella Phycodnavirus by bacterial thymidilate synthase ThyX-type
β-clip dUTPase	LGT for Orf Poxviruses, Entomopoxviruses, and Chlorella Phycodnaviruses	Probable displacement of this enzyme in the Mimivirus by a bacterial MazG dUTPase
Capping Enzyme	Basal	
ATP-dependant Ligase	LGT for phycodnavirus EHV86 and vertebrate Poxviruses	Probable displacement of the ancestral NAD dependant Ligase by this enzyme in EHV86 and Poxviruses
RNA polymerase ssu 1	Basal	
RNA polymerase ssu 2	Basal	
Topoisomerase II	Basal	Probable displacement of this enzyme in the Mimivirus and Poxviruses by a bacterial Topoisomerase I
RNA polymerase ssu 10	Basal	

- **ATP-dependent DNA ligase **(Fig. 3A) (several vertebrate Poxviruses and the Phycodnavirus EHV86). In the case of EHV86, the sequence is closely related to those belonging to the plantae lineage (plant and red/green alga). The algal host of EHV86, Emiliana huxleyi, is a haptophyte that contains a red algal endosymbiont. Thus, the virus may have recruited its ATP-dependant DNA ligase from a gene carried by the photosynthetic red algal endosymbiont. Interestingly, Mimivirus, Iridoviruses and Entomopoxviruses carry a non-homologous NAD dependant DNA ligase. The phylogeny of this version of the enzyme does not display any evidence of transfers (data not shown). It is tempting to suggest that the NAD dependant ligases are the ancestral version of the enzyme and that these have been displaced by an ATP-dependant ligase from the hosts in several NCLDV lineages (vertebrate Poxviruses and EHV86).

- **dUTPase **(Fig. 3B) (*Chlorella *Phycodnaviruses, Entomopoxviruses, amphibian Iridoviruses, and the contagious pustular dermatitis Poxviruses, also called orf Poxvirus). In the case of the dUTPase, Mimivirus uses a different version which is a homolog of the MazG type dUTPase probably acquired from bacteria (Iyer 2006).

- **Serine/Threonine Kinase **(Fig. 3C) (vertebrate Poxviruses and the CIV Iridovirus).

- **Thymidine Kinase **(Fig. 3D)(vertebrate Poxviruses and CIV Iridovirus).

- **Ribonucleotide Reductase **(Fig. 3E and 3F) (small subunit: mammalian Poxviruses and Trichoplusia ni Ascovirus; large subunit: mammalian Poxviruses).

In addition, although the folate-dependant thymidilate synthase (ThyA-type) of all NCLDV lineages is polyphyletic and scattered within the tree, all retain a distant relationship with those of the respective hosts (data not shown). This might also reflect lateral gene transfer. Interestingly, the *Chlorella *Phycodnaviruses use a flavin-dependent thymidilate synthase (ThyX-type), probably deriving from Bacteria [29], that has replaced the ancestral folate-dependant thymidilate synthase. Close examination of the phylogenies show that core genes putatively acquired from the host are a mix of recent and more ancient events. For example, among the 8 putative cases of transfers in the Poxvirus genomes, four events appears before the splits of the mammalian or the avian lineages (Fig. 3B, C and 3D). On the other hands, the four remaining events are more ancient *ie *predating the birds and mammals divergence (Fig. 3E and 3F) or predating the divergence between the deuterostomes and the protostomes (Fig 2A).

In summary, the phylogeny of the core genes involved in DNA metabolism provide evidence of frequent lateral transfers from different cellular sources to NCLDV. As noted above, Poxviruses seem the more affected by these transfers. However, we cannot rule out that these genes are not "true" NCLDV core genes but result from multiple and independent acquisition of copies from different cellular sources (host or bacterial prey of the host). Alternatively, these transfers could constitute independent homologous and non-homologous replacement of the version of the gene already present in the common NCLDV ancestor.

### Other core genes are transmitted vertically from the common ancestor of the NCLDV

Additionally, we have carried out a phylogenetic analysis of all other core genes with recognizable cellular homologs (table 1). Analysis of the different trees does not provide clear evidence of additional gene transfers among these core genes: in almost all of the phylogenies, NCLDV sequences form monophyletic groups at the base of the eukaryotic tree. The only exception could be topoisomerase II which, in the case of the Poxviruses and the Mimiviruses, has been displaced by a bacterial-like topoisomerase I [30]. Taken together these observations suggest that the number of ancestral NCLDV core genes could be smaller than previously suggested [10] because several enzymes (mainly involved in DNA metabolism) could have been acquired independently by the NCLDV lineages from their respective hosts.

### Metazoan NCLDVs show a predominant acquisition of host genes compared to Phycodnaviruses and the Mimivirus

**Figure 4:**
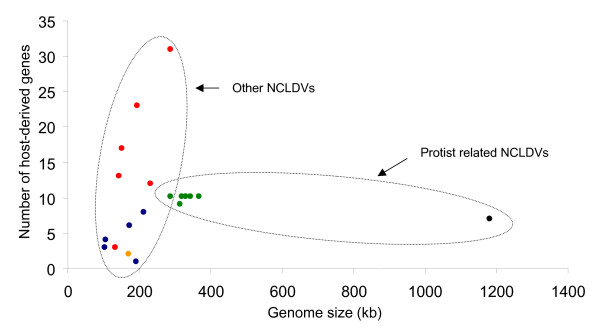
**Number of host derived genes in the NCLDVs**. The number of host derived genes for each virus is plotted as a function of their genome size. Poxviruses are indicated with a red circle, Iridoviruses are in blue, the Asfarvirus in orange, Phycodnaviruses in green and the Mimivirus in black.

Finally, we have plotted the number of genes, validated by their phylogeny, that display clear evidence of lateral transfer with their host (Fig 4). It is clear that Poxviruses and, to a lesser degree, Iridoviruses and Asfarviruses, have undergone frequent gene exchanges with their host. Fowl Poxvirus, for example, carries the largest proportion of host-derived genes of the entire NCLDV group. We also note that in general among the Metazoan NCLDV, the bigger the genome, the higher the number of host-derived genes. In contrast, *Chlorella *Phycodnaviruses and the Mimivirus, which have larger genomes, have a lower number of host-derived genes. Moreover there does not seem to be a correlation between genome size and host-derived gene abundance in these cases. This result is in striking contrast with that in Fig. 1 which shows that Metazoan viruses have fewer bacterial-like genes than the viruses which are in intimate contact with protists. This is in agreement with our previous suggestion that the *Chlorella *Phycodnaviruses and the Mimivirus derive their bacterial-like genes from the prokaryotic prey of their host (or host symbiont). We propose that, among the NCLDV, there is a general trend of acquisition of genes of cellular origin and that (at least) two distinct evolutionary pathways may have been used:

- Metazoan viruses preferentially derive their "new" genes from their host.

- Protist-infecting viruses preferentially derive their new genes from the bacterial prey of their host.

Unfortunately, the complete genome sequences of *Ectocarpus siliculosus *and *Emilinia huxleyi *are not yet available and we have not therefore been able to analyse host gene acquisition by their respective Phycodnaviruses. These viruses (EHV86: 407 kb and ESV-1: 336 kb) have similar genome sizes to other Phycodnaviruses that infect symbiotic algae but are larger than NCLDV members that infect Metazoa. As these viruses have low proportions of bacterial-type genes, we might predict that they have accumulated a large collection of eukaryotic-derived genes. Interestingly, the initial report of the genome sequence of EHV86 shows the existence of a large region of 100 kb with very few homologies with NCLDV and with genes in general databanks [31]. This region could be a hotspot for insertion of host-derived genes as observed, for example, at the tips of the Poxvirus, Mimivirus and *Chlorella *Phycodnavirus genomes.

Additionally, one can argue that these host-derived genes are in turn the results of a lateral gene transfers from a virus or a phage. In this case, a large proportion of the host-derived genes identified in this study will have homologues in viruses. For a representative set of NCLDVs, we used a BLAST approach seeded with genes identified as host-derived against a complete viral databanks purged from NCLDV sequences. Additional file 4 indicated the total number of host derived genes for each NCLDV genomes and the number of host derived genes that has viral homologues. The number of host derived genes that has viral homologues infecting the same host (or a closely related host) are also mentioned. This analysis shows that only a minor proportion of the host-derived genes have viral homologues (0–27%), and this proportion decrease significantly if we take into account only homologues of viruses infecting the same (or related) hosts (0–16%). In one case, the Iridovirus IIV6, the proportion reaches 50% but this viral genome seems to be modestly affected by gene transfers (only 8 genes were identified as deriving from the host genome). We can also note that NCLDVs genes that have viral homologues could be the results of independent gene acquisitions from their respective hosts, and thus do not constitute direct evidences of virus to virus gene transfers. In other words, this analysis probably over-estimated the level of inter-virus gene transfers because some of them are probably the results of independent genes acquisitions from the hosts. Taken together, these results show that most, if not all, of the host derived genes identified in this study do not have a viral origin and thus constitute *bona fide *evidences of host-to-virus gene transfers.

## Conclusion

In this and the previous study [27], we have shown that the large diversity of the genomic repertoire of the different lineages of NCLDV can be explained principally by the accretion of a sizeable collection of cellular genes derived from their host (notably by Metazoan NCLDV) and/or from bacteria (mainly for the viruses infecting protists and symbiotic algae). In addition, recent studies underlined the importance of lineage specific gene duplication during the evolution of the NCLDV [9,32]. For example, Mimivirus has more than 400 duplicated genes belonging to 80 different families that are generally not present outside this genome [32]. These different observations are not in accord with the idea that NCLDV, and especially the giant representatives of the family, have undergone a simplification process by loosing a large array of genes from a complex cellular ancestor [8]. Moreover, the results from this study suggest that NCLDV display a general trend of genome growth by accretion of a wide variety of genes from cellular sources. Thus, it appears unlikely that the NCLDV ancestor was a cellular organism. Moreover, the NCLDV capsid is homologous to those of Adenovirus and of some bacteriophages and archaeal viruses [33]. These arguments support a viral origin for the NCLDV, including the giant Mimivirus.

The nature and level of complexity of the NCLDV ancestor remains speculative, but comparative genomic [10] and phylogenetic analysis of conserved "core" genes of the family (this study) raises the possibility that it might have been a small DNA virus with a limited subset of 30–40 genes encoding principally DNA replication and virion structural proteins, in addition to several transcription enzymes.

The presence, in the common ancestor, of enzymes implicated in the final steps of DNA metabolism is still controversial as almost all these enzymes display clear evidence of gene transfer from their host (this study). However such transfers could have occurred only recently by homologous and non-homologous replacement of viral-type ancestral copies in NCLDV. The probable small genome size of the ancestor would be compatible with a transition from an ancient RNA virus which subsequently "invented" DNA to protect its genome from RNA-degrading or modifying enzyme of its host [19]. The existence of viruses with both RNA and DNA states, such as retroviruses or hepadnaviruses, constitutes a supplementary argument for an RNA to DNA transition as the origin of primeval DNA viruses including the NCLDV ancestor [19]. This view could explain why the replication enzymes of the NCLDV are very distantly related to their host counterparts: they would have been invented or recruited by the NCLDV ancestors very early during the RNA to DNA transitions. According to one hypothesis [19], archaeal viruses and bacteriophages may have invented their own DNA genome independently. This might provide a simple explanation for the diversity of DNA machineries of the different groups of DNA viruses. Acquisition of a stable DNA genome that is faithfully replicated would then allow these viruses to grow progressively in genome size and complexity. From this simple DNA ancestor, each NCLDV lineage could have subsequently acquired a large number of lineage-specific genes from a cellular source. Eventually this could have been followed by extensive gene duplication which would have contributed to the gigantism of several representatives of the family.

Alternatively, the NCLDV ancestor could have resulted from escape of an ancient "selfish" eukaryotic DNA element that had acquired a capsid gene from a DNA virus to become infective. The small DNA genomes of each NCLDV lineage could have undergone extensive growth in genome size and complexity. Interestingly such an intermediate between eukaryotic viruses and selfish mobile elements, the Maverick transposon, exists in a variety of eukaryotic genomes. This displays typical characteristics of mobile elements such as an integrase and long terminal inverted repeat with multiple copies in a given genome and typical features of viruses such as a protein-primed DNA polymerase and packaging or capsid protein [34]. As previously mentioned, the NCLDV capsid is related to those of bacteriophages and archaeal viruses [33], as well as the A32-like packaging ATPase which have closer homologs in eukaryotic transposons and in DNA bacteriophages. This latter enzyme is probably involved in the separation of viral chromosomes and their packaging into the virion [10]. These NCLDV core genes with no cellular homologs would favour a chimeric origin for the NCLDV ancestor between a prokaryotic DNA virus and a eukaryotic transposon [11]. However, the "chimeric DNA hypothesis" does not explain why archaeal viruses, eukaryotic viruses and bacteriophages use very different mechanisms of DNA replication.

These two hypotheses for the origin of the NCLDV ancestors ("RNA to DNA transition" and "escaped/chimeric DNA") seem to provide a more likely scenario than does the cellular reduction hypothesis since the latter is not compatible with the observed extensive gene accretion and lineage-specific gene duplication which occured subsequent to the division of each NCLDV group.

## Methods

### Sequence retrieval

A representative of each NCLDV clade was randomly chosen and the complete genome sequences were retrieved from Genbank using the following access numbers: Mimivirus (GenBank: NC_006450); Phycodnavirus: PBCV1 (GenBank: NC_000852), *Emilinia huxleyi *virus 86 (GenBank: NC_007346) and *Ectocarpus silicosus *virus 1 (GenBank: NC_002687); Poxvirus: *Amsamcta Moorei *entomopoxvirus (GenBank: NC_002520), Vaccinia virus (GenBank: NC_006998), Fowlpox Virus (GenBank: NC_002188), Lumpy skin disease virus (GenBank: NC_003027), *Molluscum contagiosum *virus (GenBank: NC_001731), Yaba-like disease virus (GenBank: NC_002642), *Melanoplus sanguinipes *entomopoxvirus (GenBank: NC_001993), Bovine popular stomatosis virus (GenBank: NC_005337); Iridovirus: *Ambystoma tigrinum *virus (GenBank: NC_005832), Invertebrate Iridescent virus 6 (GenBank: NC_003038), Infectious spleen and kidney necrosis virus (GenBank: NC_003494), Lymphocystis disease virus 1 (GenBank: NC_001824), *Aedes taeniorhynchus *iridescent virus (GenBank: NC_008187), *Trichoplusia ni *ascovirus (GenBank: NC_008518); Asfarvirus: African Swine Fever virus (GenBank: NC_001659). Additional Phycodnaviruses AR158, NY2A, FR483, MT325 and ATCV1 were downloaded from Greengene at http://greengene.uml.edu/database/database.html.

### Dataset construction

The phylogenetic affinities of each NCLDV ORF were determined using BLASTP against a non-redundant database with an exclusion threshold of *E *< 10^-5 ^[35]. If the query produced genes from different kingdoms (Eukarya/Bacteria/Archaea/Virus) within the first ten hits of a BLAST search, the evolutionary status of the gene was analysed further by individual phylogenetic analysis (see below). If the queries retrieved matches with a single kingdom within the first ten hits, the affinities of the gene were assigned directly. This approach previously allowed us to identify a large proportion of bacterial-type genes in Giant NCLDV [27]. This analysis also revealed the presence of a large panel of genes with Eukaryotic affinities that are putative candidates for lateral gene transfer with the host. However, phylogenetic analysis of each gene is time consuming and difficult to automate. Fortunately, most of the host (or related) genomes are now available and we could therefore search for viral genes that are closely related to their host gene homologues.

To specifically identify candidates of LGT we proceeded as follows: for each ORF with eukaryotic affinities, we determined all the reciprocal best matching ORFs between the virus and its host (or, when unavailable, closely related organisms) and then between the virus and a Non-Redundant (NR) database purged of host or closely related sequences. For example, for the Fowlpox virus we used BLAST to compare each ORF with eukaryotic affinities against the *Galus galus *genome. This was then repeated against a NR database purged of all vertebrate sequences. The BLAST score expressed as a BIT score http://www.ncbi.nlm.nih.gov/BLAST/tutorial/Altschul-1.html of each viral ORF against the *G. galus *data set was then plotted against those obtained from the non-vertebrate set (Fig. 2). Those viral genes having a higher affinity with *G. galus *fall below the diagonal. Additionally the ratio of the *G. galus *BLAST score and that of the non-vertebrate BLAST score was plotted against the position of the ORF within the viral genome. In this case, points which occur above the horizontal line (Fig. 2) are more closely related to the host than to other non-vertebrate species. This supplies important information concerning gene position and linkage on the genetic map. For the bacterial-type genes, this provided evidence of gene co-capture [27]. Finally, all good candidate ORFs were validated using individual phylogenetic analysis.

To identify viral homologs of the host-derived genes identified before, we BLAST each of these ORFs against a Viral Database purged from the NCLDVs sequences using a exclusion threshold of *E *< 10^-5 ^. Each positive searches were divided into two categories : (1) NCLDV sequences that match with sequences from viruses infecting the same host or a closely related host and (2) NCLDV sequences that match with sequences from viruses infecting a different host.

### Statistical tests

Statistical analyses were performed using the reciprocal ratio, R, (BLAST score NR database/BLAST score host genome) to avoid infinite values. ORF positions were converted to a relative distance, D, from the genome centre. The plots of this ratio as a function of the relative ORF position on the genome, R = f(D), illustrate the distribution along the host genome. To confirm the visual effect, we fitted the profile to an empirical function: R = 1 + A*D^2 ^using GraphPad Prism v4.0. For each dataset, we performed two fits, with the parameter A either unconstrained (non-random distribution of ORFs along the genome) or constrained to 0 (no dependence of the relative ORF position). Results were compared with an F test (*P *> 0,05) to determine the best fit.

### Phylogenetic analysis

Homologous sequences were identified by BLAST and a representative set of viral and eukaryotic sequences were randomly chosen to cover all the recognized lineages of each group of NCLDV (Poxvirus, Phycodnavirus etc...). These alignments were inspected and manually refined. Gaps and ambiguously aligned positions were eliminated from the phylogenetic analysis. For all markers, we performed preliminary analyses by NJ (without correction) using MUST 3.0 [36] by Maximum likelihood (ML), using PROML (phylip v3.6) with the JTT amino acid substitution matrix, a rate heterogeneity model with gamma-distributed rates over four categories with the α parameter estimated using TREE-PUZZLE, global rearrangements and randomized input order of sequences (10 jumbles).

## Authors' contributions

JF conceived the project created the datasets and performed the computational analysis, NP built the statistical tests and MC supervised the work. JF, NP and MC wrote the manuscript.

## Supplementary Material

Additional File 1 **Genomic map of Mimivirus and *Chlorella *Phycodnaviruses.** The putative phylogenetic origins of the genes are indicated with the following colours: red corresponds to bacterial type genes, blue to eukaryotic genes, green to NCDLV genes and black to the orphan genes. The orphan genes are placed below on the genomic map. The positions of the IS*607 *elements are indicated by a red arrow. The Mimivirus (1.2 Mb) and Phycodnaviruses (300–400 Kb) are not to the same scale. The intervals under the genomic map represent 100 Kb. Click here for file

Additional File 2 **Number of genes in the NCLDVs that have host homologs.** The number of NCLDV genes that have host homologs is plotted with respect to their genome size. Poxviruses are indicated with red circles, Iridoviruses are in blue, the Asfarvirus in orange, Phycodnaviruses in green and the Mimivirus in black. The corresponding coloured lines indicate the regression line of the Poxviruses (red), of the Iridovirus (blue) and of the Mimivirus and *Chlorella *phycodnaviruses (green). Click here for file

Additional File 3 **Ratio R, (NR database BLAST score/host BLAST score) as a function of the ORF relative distance D to the genome centre.** Data were fitted by the empirical relationship: R = 1 + A*D^2^, with or without constraining A to equal 0 random distribution or clustered repartition). The best fit value was chosen with an F test, *P *< 0,05, (represented in solid line). For the dotted line, parameter A is equal to 0, representing independence between R and D. Click here for file

Additional File 4 **Number of host derived genes in the NCLDVs that have homologues in other groups of viruses.** For a representative subset of NCLDV genomes, we have plotted the total numbers of host-derived genes (black circles). Among these host- derived genes, those with homologues in other viral genomes are indicated with white circles. Among these viral homologues, those with homologues in viral genomes that infect the same or a closely related host are indicated with grey circles. Click here for file
