# Supplementary material for: Phylogenetic evidence for extensive lateral acquisition of cellular genes by Nucleocytoplasmic large DNA viruses
Source: BMC Evol Biol. 2008 Nov 26;8:320. doi: 10.1186/1471-2148-8-320 (PMC2607284; doi:10.1186/1471-2148-8-320)
Supplement: Additional File 1 — Genomic map of Mimivirus and Chlorella Phycodnaviruses. The putative phylogenetic origins of the genes are indicated with the following colours: red corresponds to bacterial type genes, blue to eukaryotic genes, green to NCDLV genes and black to the orphan genes. The orphan genes are placed below on the genomic map. The positions of the IS607 elements are indicated by a red arrow. The Mimivirus (1.2 Mb) and Phycodnaviruses (300–400 Kb) are not to the same scale. The intervals under the genomic map represent 100 Kb. [file 1471-2148-8-320-S1.ppt]

## Slide 1
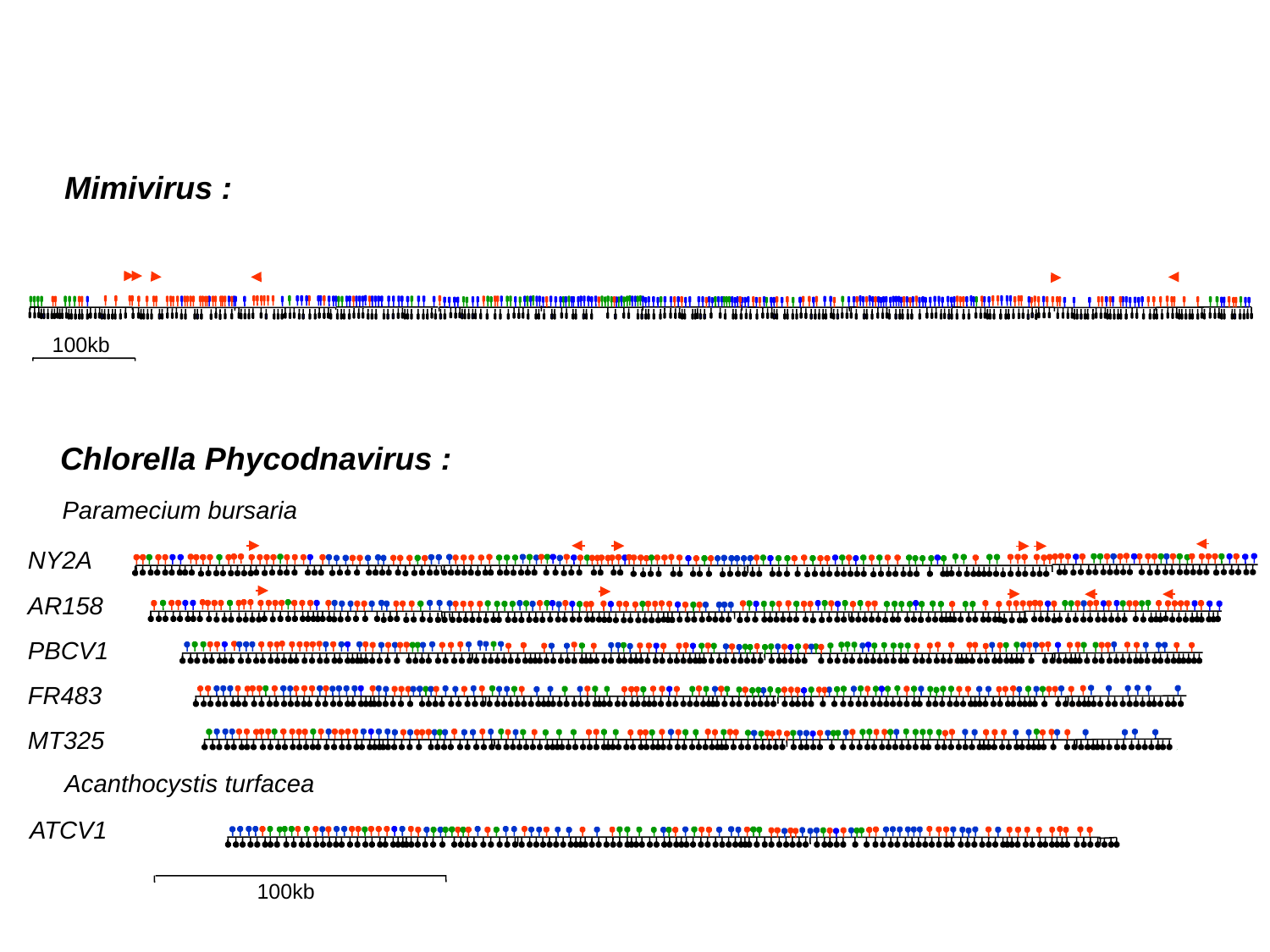

Mimivirus :
100kb
Chlorella Phycodnavirus :
Paramecium bursaria
NY2A
AR158
PBCV1
FR483
MT325
Acanthocystis turfacea
ATCV1
100kb
