# Supplementary figures and images for: Phylogenetic evidence for extensive lateral acquisition of cellular genes by Nucleocytoplasmic large DNA viruses
Source: BMC Evol Biol. 2008 Nov 26;8:320. doi: 10.1186/1471-2148-8-320 (PMC2607284; doi:10.1186/1471-2148-8-320)

## Slide 1
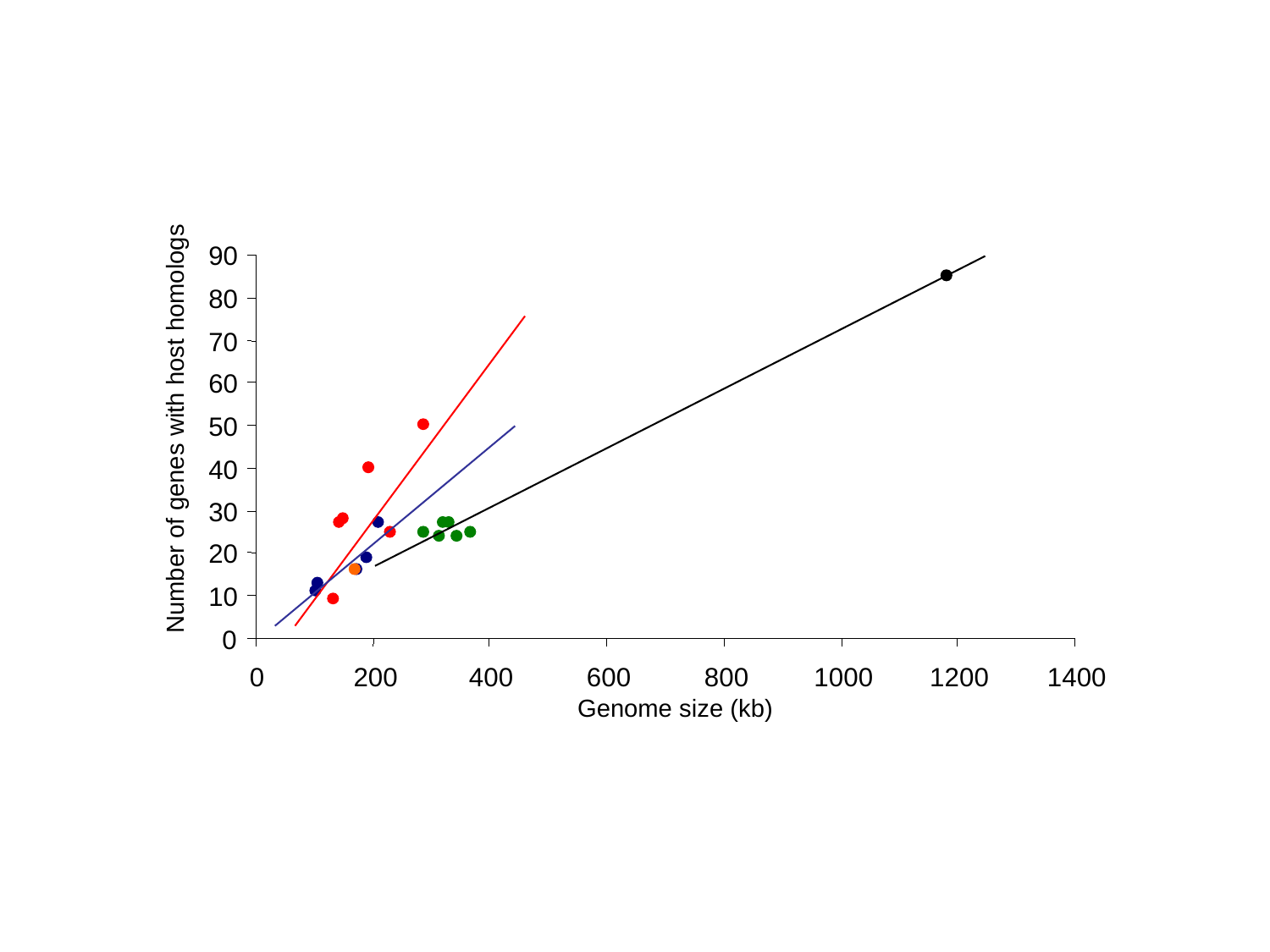

90
80
70
60
Number of genes with host homologs
50
40
30
20
10
0
0
200
400
600
800
1000
1200
1400
Genome size (kb)

Supplement: Additional File 2 — Number of genes in the NCLDVs that have host homologs. The number of NCLDV genes that have host homologs is plotted with respect to their genome size. Poxviruses are indicated with red circles, Iridoviruses are in blue, the Asfarvirus in orange, Phycodnaviruses in green and the Mimivirus in black. The corresponding coloured lines indicate the regression line of the Poxviruses (red), of the Iridovirus (blue) and of the Mimivirus and Chlorella phycodnaviruses (green). [file 1471-2148-8-320-S2.ppt]

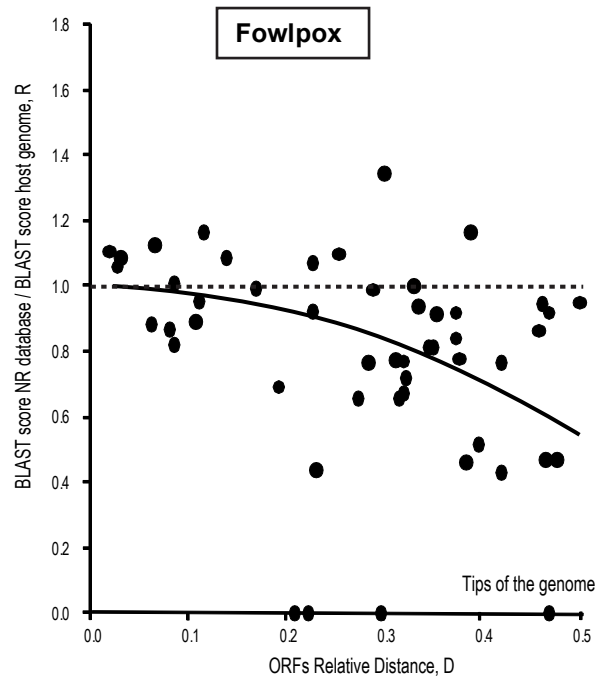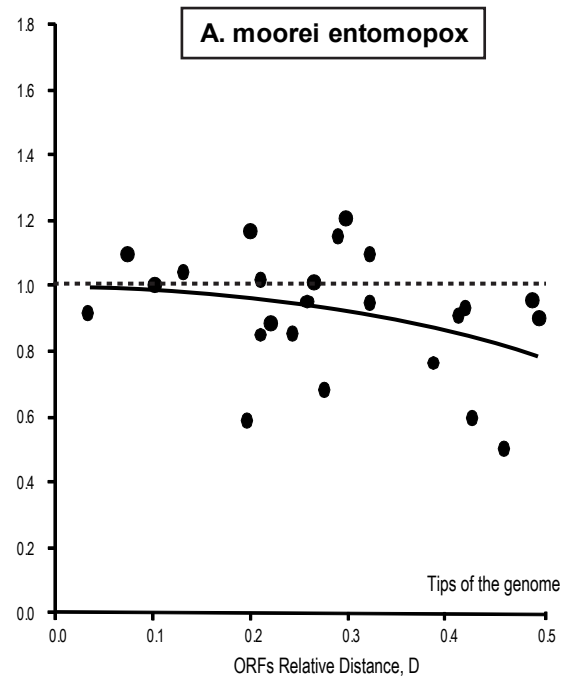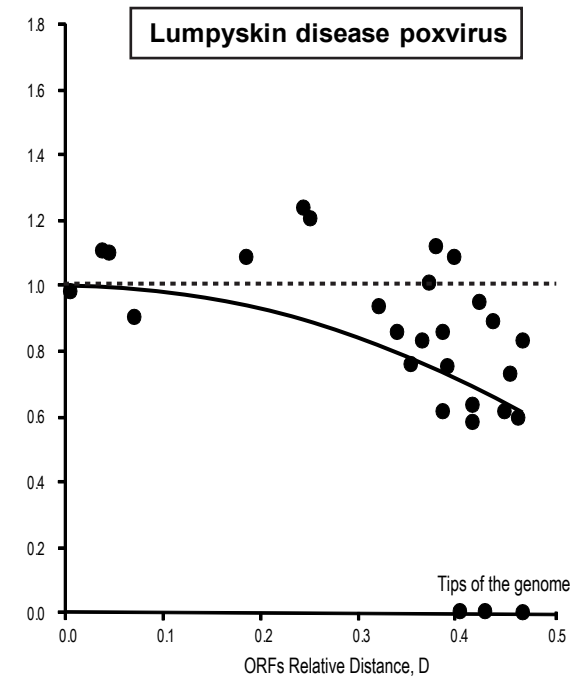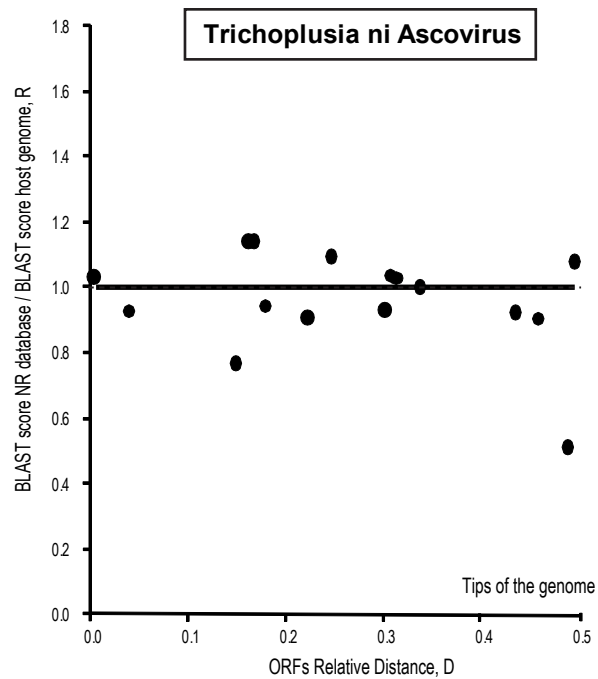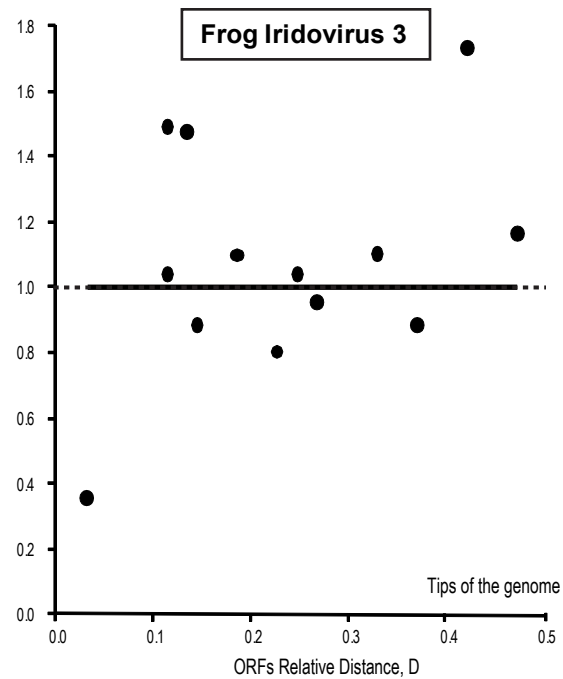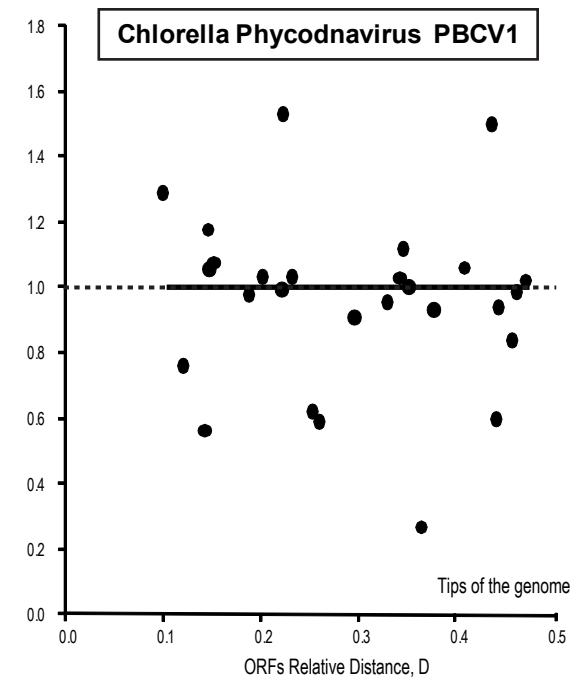

Supplement: Additional File 3 — Ratio R, (NR database BLAST score/host BLAST score) as a function of the ORF relative distance D to the genome centre. Data were fitted by the empirical relationship: R = 1 + A*D2, with or without constraining A to equal 0 random distribution or clustered repartition). The best fit value was chosen with an F test, P < 0,05, (represented in solid line). For the dotted line, parameter A is equal to 0, representing independence between R and D. [file 1471-2148-8-320-S3.pdf]

## Slide 1
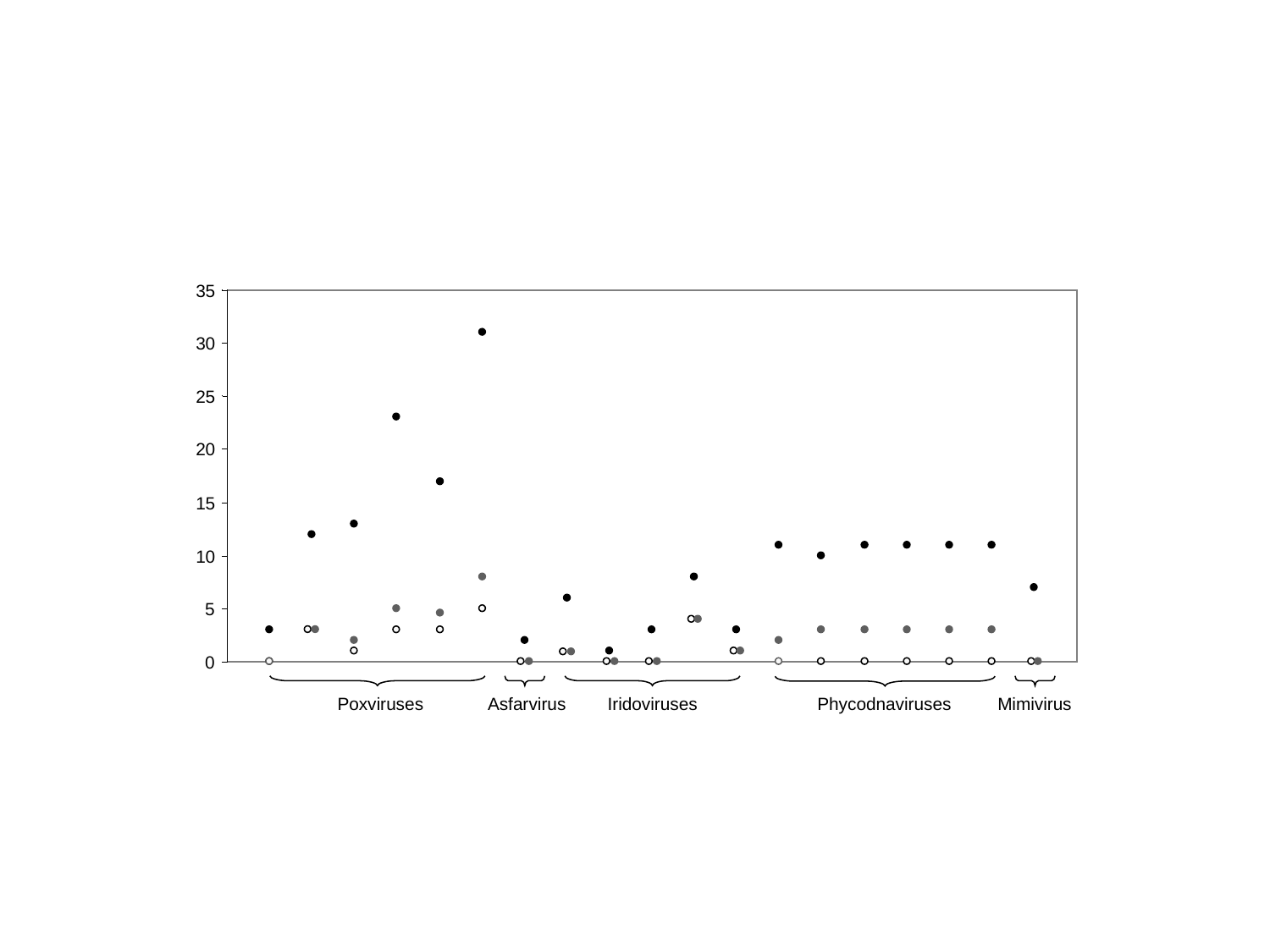

35
30
25
20
15
10
5
0
Poxviruses
Asfarvirus
Iridoviruses
Phycodnaviruses
Mimivirus

Supplement: Additional File 4 — Number of host derived genes in the NCLDVs that have homologues in other groups of viruses. For a representative subset of NCLDV genomes, we have plotted the total numbers of host-derived genes (black circles). Among these host- derived genes, those with homologues in other viral genomes are indicated with white circles. Among these viral homologues, those with homologues in viral genomes that infect the same or a closely related host are indicated with grey circles. [file 1471-2148-8-320-S4.ppt]
